# Editorial: Advances in Food Metabolomics for Functional Food Development and Analysis

**DOI:** 10.3390/metabo16060396

**Published:** 2026-06-07

**Authors:** M. J. Pilar Martinez-Martin, Amanda J. Lloyd

**Affiliations:** Department of Life Sciences, Aberystwyth University, Aberystwyth SY23 3DA, UK; abl@aber.ac.uk

## 1. Introduction

The increasing global demand for healthier diets and functional foods has positioned food metabolomics at the forefront of nutritional science and food innovation. By comprehensively characterising small molecules in food systems, metabolomics provides detailed insights into food composition, processing effects, and the biological significance of dietary components.

Metabolomics offers a robust approach for analysing the interplay between food composition and biological function. Generating comprehensive metabolic profiles enables the identification of bioactive compounds, the evaluation of their processing stability, and the assessment of their bioavailability and physiological impact. In addition, metabolomics facilitates the discovery of biomarkers of dietary intake and metabolic responses, supporting advances in precision nutrition and, increasingly, personalised dietary strategies.

This Special Issue, *“Food Metabolomics: Bioactive Compounds and Functional Food Development,”* presents multidisciplinary research on how metabolites affect food quality, bioavailability, and human health. The studies highlight advances in analytical technologies, experimental models, and translational approaches supporting evidence-based functional food development.

## 2. Advances in Characterising Bioactive Food Metabolites

A central focus of this Special Issue is the identification and functional characterisation of bioactive compounds in foods. Li et al. demonstrated that tissue-specific metabolomics of *Ampelopsis grossedentata* revealed significant variation in flavonoid content and antioxidant capacity (Contribution 1). Floral tissues, in particular, have emerged as a promising resource for functional food development due to their enriched flavonoid and amino acid profiles, highlighting the importance of targeted harvesting strategies.

Similarly, Warren-Walker et al. investigated the impact of spray drying on the chemical composition of ginger (Contribution 2). Their findings demonstrate that processing conditions and carrier selection influence the formation and preservation of key bioactives, such as gingerols and shogaols, highlighting the balance between technological processing and nutritional quality. Together, these studies show how both biological variability and processing conditions shape the metabolomic landscape of foods, reinforcing the importance of integrating compositional analysis with food processing strategies in the development of functional products.

## 3. Metabolomics in Understanding Diet–Health Relationships

Beyond food composition, metabolomics plays a critical role in linking dietary intake to health outcomes. Fernández-Cruz et al. identified urinary hippuric acid as a sex-dependent biomarker of fruit and nut intake, demonstrating the potential of metabolomics to provide objective tools for dietary assessment and to support precision nutrition approaches (Contribution 3). This work highlights the importance of considering biological variability, including sex-specific differences, when interpreting metabolomic data in nutritional studies.

In a complementary human intervention study, Cooper et al. compared the metabolic effects of cottonseed oil and olive oil. Although global metabolomic profiles were broadly similar, distinct differences in immunoregulatory lipid mediators were observed, indicating that dietary fats can differentially influence inflammatory pathways and immune responses (Contribution 4). These findings provide important mechanistic insight into how dietary lipid composition may modulate metabolic and inflammatory processes in humans. Together, these studies underscore the capacity of metabolomics to move beyond traditional dietary assessment methods by enabling objective measurement of dietary exposure and revealing mechanistic links between diet and health outcomes.

## 4. Emerging Approaches to Evaluate Bioactivity and Mechanisms

Understanding the systemic effects of bioactive compounds remains a significant challenge in food and nutritional science. Peron et al. utilised an untargeted urinary metabolomics approach to investigate the effects of *Betula alba* extract in a preclinical model (Contribution 5). While no direct diuretic effect was observed, significant metabolic alterations were detected, indicating broader physiological activity. In particular, changes in corticosteroid metabolism and bile acid pathways suggest potential interactions with endocrine regulation, hepatic function, and gut microbiota-related processes.

These findings highlight the sensitivity of metabolomics in detecting subtle biochemical changes that may not be captured through conventional physiological measurements. More broadly, they reinforce the value of untargeted metabolomics as a hypothesis-generating tool for uncovering unexpected bioactivities and advancing our understanding of the mechanisms underlying the health effects of bioactive compounds.

## 5. Integrating Analytical Platforms and Experimental Models

The Special Issue illustrates how combining advanced analytical platforms with robust experimental systems enhances metabolite profiling and research reliability.

Such integrative approaches are increasingly supported by advanced infrastructure, including high-resolution metabolomics platforms capable of analysing complex biological matrices and identifying biomarkers in both food and human samples. Complementary in vitro digestion systems enable controlled simulation of gastrointestinal processes to assess nutrient bioavailability and transformation.

Specialised units also enable quantitative assessment of protein digestibility and amino acid composition, providing critical insights into nutritional quality. These analytical capabilities are integrated with human intervention trials and community-based research frameworks, facilitating the translation of metabolomics findings into real-world dietary and health outcomes. The convergence of advanced analytical precision, experimental modelling, and human studies is essential for advancing functional food research.

Increasingly, these integrated analytical frameworks are being applied to the reformulation and evaluation of staple foods with the aim of improving nutritional quality and metabolic health outcomes. Cereal-based products such as bread represent an important target for metabolomics-guided innovation due to their widespread dietary contribution and their relevance to glycaemic response, fibre intake, and overall dietary quality. Emerging translational initiatives are combining metabolomic profiling with digestion modelling, ingredient functionality assessment, and bioavailability studies to better understand how processing and formulation influence the generation and stability of health-relevant metabolites. Such approaches are exemplified by collaborative programmes, including the Innovate UK-supported Better Bread initiative, which aim to integrate analytical science, food technology, and nutritional research to support the development of healthier staple food products with demonstrable functional potential.

## 6. Future Perspectives

The contributions to this Special Issue identify several emerging priorities for the field. Standardising metabolomic workflows is essential to improve reproducibility and comparability across studies. Integrating multi-omics approaches, including genomics, transcriptomics, and proteomics alongside metabolomics, will further clarify complex food–health interactions by linking dietary components to underlying molecular mechanisms and physiological responses. There is also increasing emphasis on developing metabolite biomarkers as practical tools for dietary assessment and, more broadly, for advancing precision nutrition. In parallel, these innovations must align with consumer expectations and regulatory requirements, particularly regarding clean-label products and substantiated health claims. As food metabolomics continues to advance, collaboration among food scientists, nutritionists, analytical chemists, and policymakers will be critical to translating scientific progress into meaningful improvements in public health.

## 7. Conclusions

This Special Issue highlights the transformative role of metabolomics in food science, from characterising bioactive compounds and understanding processing effects to elucidating diet–health relationships and supporting the development of next-generation functional foods. The studies presented collectively demonstrate how metabolomics is increasingly contributing not only to analytical characterisation, but also to translational food innovation, including the reformulation and optimisation of staple foods aimed at improving public health outcomes. We thank all authors for their valuable contributions and the reviewers for their critical insights. We hope this collection will inspire further research that bridges the gap between food composition, biological function, and public health impact.

